# InSituPy: a framework for histology-guided, multi-sample analysis of single-cell spatial omics data

**DOI:** 10.1093/bioinformatics/btag073

**Published:** 2026-02-15

**Authors:** Johannes Wirth, Anna Chernysheva, Birthe Lemke, Isabel Giray, Katja Steiger

**Affiliations:** Institute of Pathology, School of Medicine and Health, Technical University of Munich, Munich, Germany; Institute of Pathology, School of Medicine and Health, Technical University of Munich, Munich, Germany; Institute of Pathology, School of Medicine and Health, Technical University of Munich, Munich, Germany; Institute of Pathology, School of Medicine and Health, Technical University of Munich, Munich, Germany; Institute of Pathology, School of Medicine and Health, Technical University of Munich, Munich, Germany; German Cancer Consortium (DKTK) Partner Site Munich, Munich, Germany

## Abstract

**Motivation:**

Spatial omics data provides unprecedented insights into disease biology, yet its complexity introduces significant challenges in data analysis. Comprehensive analysis requires frameworks that integrate diverse modalities and enable joint processing of multiple datasets and corresponding metadata.

**Results:**

To address these challenges, we introduce InSituPy, a versatile and scalable framework for analyzing spatial omics data from the multi-sample level down to the cellular and subcellular level. Its hierarchical data structure organizes all relevant data modalities per sample and links them to their corresponding metadata, enabling scalable analysis of large patient cohorts using spatial omics technologies. Interactive visualization tools within InSituPy enable seamless integration of histopathological expertise, promoting collaborative hypothesis generation in translational research. Additionally, InSituPy includes built-in analytical algorithms and interfaces with external tools, establishing a standardized workflow for multi-sample spatial omics data analysis.

**Availability:**

The Python package InSituPy is publicly available on GitHub (https://github.com/SpatialPathology/InSituPy) and PyPi (https://pypi.org/project/insitupy-spatial/), and archived on Zenodo (DOI: 10.5281/zenodo.18459471). Tutorials and documentation for InSituPy are available at https://insitupy.readthedocs.io/. All code to replicate the results shown in this manuscript can be found in the GitHub repository. Scripts to connect QuPath and InSituPy can be found at https://github.com/SpatialPathology/InSituPy-QuPath. All data required to complete the tutorials is publicly available, and functions to download the data have been implemented. A Zulip community chat for user support and discussion is accessible at https://insitupy.zulipchat.com.

**Contact:**

j.wirth@tum.de, katja.steiger@tum.de

## 1 Introduction

The emergence of spatially resolved multi-omics technologies has the potential to revolutionize our understanding of biological processes in healthy and diseased tissue. While early methods such as *Visium* (Ståhl *et al.* 2016) and *Slide-seq* (Rodriques *et al.* 2019) measured transcriptomes within micron-sized spatial units and failed to achieve single cell resolution, the development of multiplexed fluorescence in situ hybridization (multiplexed *FISH*) (Lubeck *et al.* 2014; Xia *et al.* 2019) and in situ sequencing (*ISS*) (Ke *et al.* 2013; Lee *et al.* 2014) allowed researchers to map individual RNA molecules at subcellular resolution and thus measure the transcriptional state of single cells within tissue sections. The non-destructive nature of multiplexed FISH and ISS technologies allows the combination of transcriptomic readouts with conventional image-based readouts such as histological or immunofluorescence stainings. Access to histological data enables full integration of pathological expert knowledge from routine diagnostics and opens new ways for hypothesis generation. Further, recent technologies such as *Xenium in Situ* (10X Genomics) and *MERSCOPE* (Vizgen) allow the analysis of multiple tissue sections at once, increasing the throughput of the methods and enabling the generation of large clinical cohorts, e.g. using tissue microarrays (TMAs). To fully exploit the potential of such datasets, it is important to integrate experiment-level information (e.g. clinical metadata or treatment conditions) with the single-cell spatial transcriptomics (scST) and proteomics (scSP) data. While *Python*-based frameworks such as *SpatialData* (Marconato *et al.* 2025) allow an integration of spatial omics data modalities, they do not offer a way to structurally integrate data from multiple tissue sections or tissue microarrays with its corresponding metadata. Other tools such as *ATHENA*, *MoleculeExperiment* or *SpatialExperiment* provide strategies to integrate multiple samples but lack support for different aspects of the analysis, including integration of annotations, single transcript information, or interactive visualization, or are only available in R (Martinelli and Rapsomaniki 2022; Righelli *et al.* 2022; Peters Couto *et al.* 2023). Additionally, since a growing number of researchers use spatial omics technologies in their projects, an easy usability, also for non-bioinformaticians, is key. In this publication, we present *InSituPy*, a Python-based framework to explore and analyze both single-cell and sequencing-based spatial omics data while simplifying and accelerating the preprocessing of multiple large datasets in parallel. Using published spatial transcriptomics datasets, we introduce workflows to perform multi-sample data analysis and integrate pathological expert knowledge into the analysis.

## 2 Results

### 2.1 Overall structure of the framework

**Figure 1:**
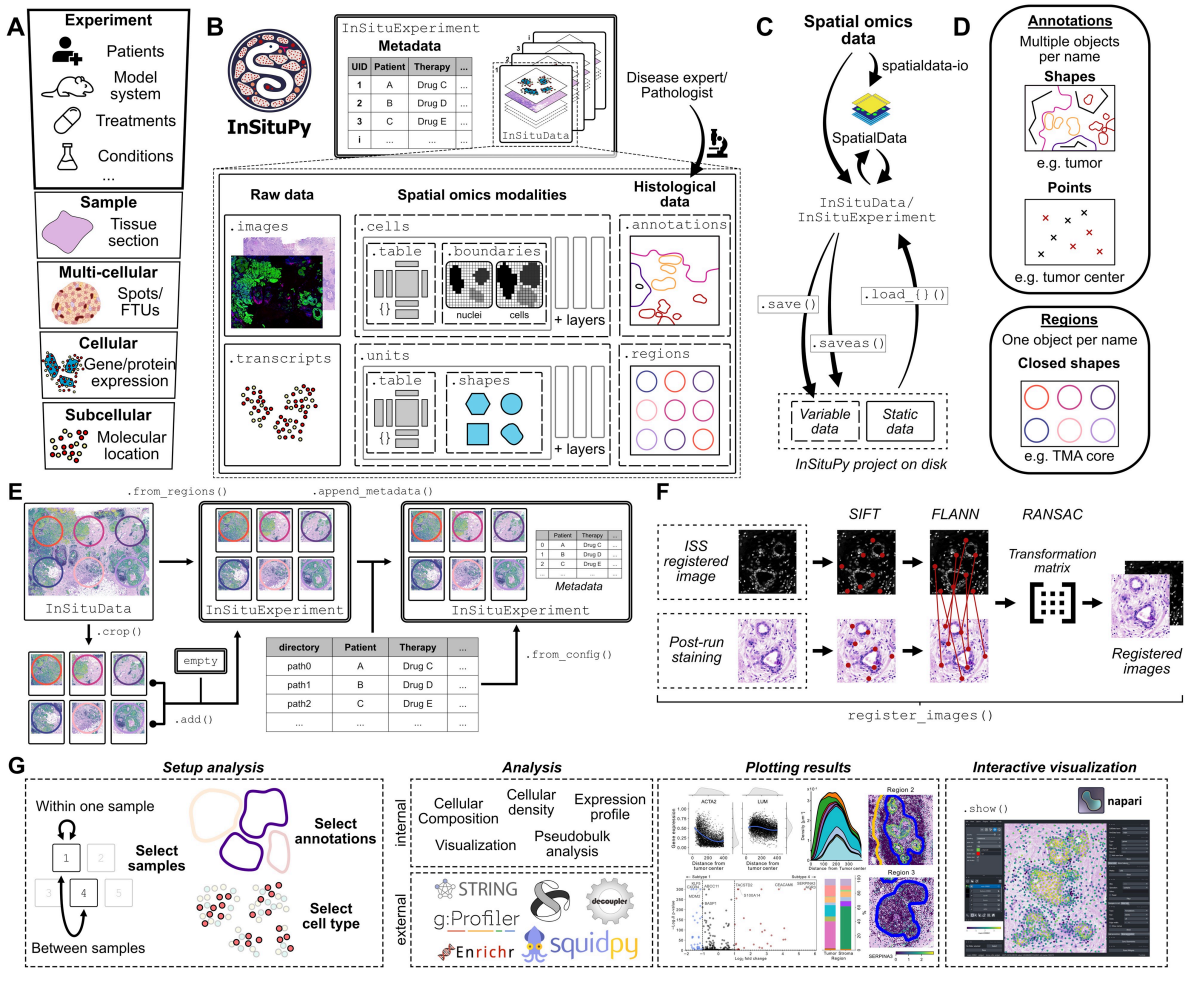
Overview of InSituPy package structure and functionalities. (A) Data levels in spatial omics datasets. (B) Experiment- and sample-level data structure of the InSituExperiment and the InSituData classes. The InSituExperiment object stores multiple datasets with their corresponding metadata while the InSituData object structures data modalities into a biologically meaningful hierarchy. (C) Reading and writing streams within the InSituPy framework. Data can be loaded directly or via the spatialdata-io ecosystem (Marconato *et al*. 2025). Data can be saved using the saveas function. On disk, the data is grouped into variable and static data. Variable data can be updated during analysis using the save function while static data remains unchanged to shorten writing times. Data modalities can be loaded separately using distinct load functions. (D) Schematic showing how histological annotations are grouped into annotations and regions. (E) Different possibilities to generate an InSituExperiment object. (F) Automated image registration workflow for aligning images of histological stainings acquired after the run with a fluorescent image that is already registered to the omics data. (G) Schematic showing possibilities to create multi-sample analysis workflows in InSituPy. Multiple internal analyses are available as well as interfaces connecting InSituPy to external analysis packages. Results can be plotted statically or visualized interactively through a napari-based viewer (Sofroniew *et al*. 2024).

Single-cell spatial omics datasets consist of multiple data levels (Fig. 1A). These levels comprise (i) the experiment level, which can contain information about the patients, the model system or treatments; (ii) the sample level, containing all data of one data entity, e.g. one tissue section; (iii) a multi-cellular level with method-specific readouts (e.g. gene or protein expression) per spatial unit; (iv) the cellular level containing the readout per individual cell; and (v) the subcellular level including molecular information such as the location of individual transcripts. InSituPy offers a hierarchical and biologically meaningful data structure to read, analyze and store data from all those five levels (Fig. 1B). Divided into two main data objects, *InSituExperiment* and *InSituData*, the framework facilitates the integration of data from multiple tissue sections. An InSituExperiment object can store multiple InSituData objects, with each InSituData object containing the raw data, spatial omics modalities and, optionally, histological information of an individual sample (Fig. 1B). Each InSituData object gets assigned a unique ID (UID) to connect the datasets with its corresponding metadata, allowing different experiment-level operations, e.g. querying datasets based on their metadata, iterating through datasets or concatenating data from multiple experiments or visualization of individual datasets (Fig. S1A-I, available as supplementary data at *Bioinformatics* online). Further, information can be transferred between InSituExperiment objects and AnnData objects, allowing the creation of analysis loops between InSituPy and the scverse ecosystem, e.g. for batch correction (Fig. S1F, available as supplementary data at *Bioinformatics* online). InSituPy is the first spatial omics analysis framework to introduce such a hierarchical data structure allowing both the handling of multiple datasets and a comprehensive analysis on the individual sample level as well as interactive visualization of the data (Table S1, available as supplementary data at *Bioinformatics* online).

### 2.2 Data structure on sample level

The sample level data structure of InSituPy (*InSituData*) is compatible with any spatially resolved omics data consisting of a count or abundance matrix, image data and optionally segmentation masks or transcript locations. Within an InSituData object, data is organized in six data categories: image data (*images*), transcript locations and identities (*transcripts*), omics data from arbitrarily shaped spatial units such as spots or tissue compartments (*units*), single-cell omics data (*cells*), histological annotations (*annotations*), and histological regions (*regions*) (Fig. 1D). While spatial units and cells both represent bounded areas with associated measurements, cells are treated independently as they possess unique properties including distinct nuclear and cellular boundaries. Both the *cells* and *units* attributes are further subdivided into layers, allowing integration of results from multiple cell segmentation algorithms or different types of spatial units. Each layer consists of two attributes: a table containing spatial omics data as an AnnData (Virshup *et al.* 2021) object, paired with either a boundaries attribute storing nuclear and cellular segmentation masks (for *cells*) or a shapes attribute containing polygons (for *units*). Each data modality can be loaded individually using the respective loading functions (Fig. 1C). Image and transcript data is loaded lazily and only loading times for cellular data, annotations and regions increase with dataset size (Fig. S2A, available as supplementary data at *Bioinformatics* online). Analysis results can be saved in an InSituPy project folder. To optimize writing performance, the framework distinguishes between *static data* (i.e. data that remains unchanged during analysis such as raw image data and transcript locations) and *variable data (i.e.* data that is modified during analysis such as spatial omics data and histological data). When saving to an existing project, only variable data is written to disk, significantly reducing save times (Fig. S2B, available as supplementary data at *Bioinformatics* online). For data handling, the framework uses different state-of-the-art packages (Table S2, available as supplementary data at *Bioinformatics* online). In case of histological data, InSituPy differentiates between regions and annotations (Fig. 1B and D). Annotations can be any histological annotation (e.g. “tumor”), while regions are meant to delineate the positions of TMA cores or different tissue sections within the same dataset.

### 2.3 Multi-sample and multi-modal data assembly

InSituExperiment objects can be assembled using three strategies (Fig. 1E): (i) from histological regions, e.g. TMA cores; (ii) from individual InSituData objects; (iii) from a configuration file containing data directories and corresponding metadata. For transcriptomic methods such as Xenium In Situ and Visium, dedicated reading functions and alignment tools are implemented, facilitating the joint analysis of single-cell and spot-based spatial omics approaches (Fig. S3A, available as supplementary data at *Bioinformatics* online). Further, technology-agnostic import workflows are documented, enabling integration of additional platforms.

For spatial proteomic technologies based on multiplex immunofluorescence (e.g. co-detection by indexing (Schürch *et al.* 2020)), a technology-agnostic workflow using QuPath (Bankhead *et al.* 2017) and InstanSeg (Goldsborough *et al.* 2024) has been implemented. This workflow enables import of one or more samples into InSituPy (Fig. S3B, available as supplementary data at *Bioinformatics* online). In addition, functionality to quantify immunofluorescence signal intensity per cell from aligned images has been implemented as well (Fig. S3C, available as supplementary data at *Bioinformatics* online). The quantification algorithm processes images in tiles, avoiding full image loading into memory and enabling analysis on standard hardware with limited RAM (Fig. S3D, available as supplementary data at *Bioinformatics* online).

### 2.4 Interoperability with SpatialData

To ensure interoperability with analysis packages from the scverse ecosystem (Virshup *et al.* 2023), InSituPy provides bidirectional conversion functions between InSituData or InSituExperiment objects and the SpatialData format (Fig. S4, available as supplementary data at *Bioinformatics* online). While SpatialData’s flat, keyword-based structure offers flexibility, it can become unwieldy when working with complex, multi-modal datasets. InSituPy addresses this limitation through a hierarchical data structure that provides organized access to different data modalities and improves scalability for larger datasets. In addition, the conversion functions provide access to *spatialdata-io* readers, extending InSituPy’s compatibility to technologies beyond its native readers. Benchmarking the InSituPy Xenium reader against its spatialdata-io counterpart revealed that spatialdata-io required substantially longer reading times and higher memory consumption, making it difficult to load very large datasets on normal computers and highlighting the advantage of technology-specialized readers over more generalized ones.

### 2.5 Automated image registration

The non-destructive nature of spatial omics methods such as Xenium or *CODEX* (Black *et al.* 2021) enables the subsequent histological staining of sections. To simplify integration of pathological annotations, InSituPy provides an automated registration pipeline that aligns the nuclear image acquired during the spatial omics measurement with subsequently stained images (Fig. 1F). The pipeline employs *Scale-Invariant Feature Transform* (SIFT) to identify features in both images (Lowe 2004), followed by the *Fast Library for Approximate Nearest Neighbors* (FLANN) for feature matching (Muja and Lowe 2009). The *random sample consensus algorithm* (RANSAC) then selects the most robust feature matches to calculate an affine or perspective transformation matrix for image registration. Registered images are saved alongside quality control metrics and integrated into the InSituData object for downstream analysis.

### 2.6 Data visualization and annotation using the InSituPy viewer

A precise registration of the images is a prerequisite for the addition of histological annotations to the datasets and for visualizing the results of the single-cell spatial omics analyses in their histopathological context. This is particularly important for morphological quality control and for leveraging the translational potential of spatial omics analyses. Histological annotations can be either imported from external software such as QuPath (Bankhead *et al.* 2017) or from a napari-based viewer (Sofroniew *et al.* 2024) (Fig. S5, available as supplementary data at *Bioinformatics* online). The viewer provides various functionalities for data examination, including visualization of spatial omics data and cellular boundaries, localization of specific cells, and adding and displaying annotations or regions (Figs. S5 and S6, available as supplementary data at *Bioinformatics* online). Added regions or annotations can be synchronized with the source InSituData object via a dedicated button. In case of multi-sample datasets stored as InSituExperiment object, individual datasets can be visualized and annotated in parallel.

### 2.7 Data analysis using InSituPy

Leveraging the InSituExperiment object structure, InSituPy facilitates multi-sample analysis workflows through simple and streamlined syntax. Analyses can be performed within or between samples, and depending on the biological question, can be focused on specific regions, annotations, or cell types (Fig. 1G). Currently available analyses include cellular composition evaluation, the measurement of cell type densities, differential gene expression analysis and pseudobulk analysis. Further, InSituPy offers interfaces to external tools such as *STRING, Enrichr* and *g:Profiler* for GO term enrichment analysis, *decoupler* for enrichment analysis, and *squidpy* for spatial analyses (Kuleshov *et al.* 2016; Raudvere *et al.* 2019; Szklarczyk *et al.* 2019; Badia-i-Mompel *et al.* 2022; Palla *et al.* 2022). Since omics data is stored in the AnnData format, all *scverse* functions are expected to be compatible with InSituPy. Further, InSituPy provides plotting functionalities to visualize the results of multi-sample datasets (Fig. S1H, available as supplementary data at *Bioinformatics* online). Together, these functionalities allow a histomorphology-informed analysis of gene expression changes in multi-sample spatial omics datasets. A complete step-by-step analysis has been demonstrated using published breast cancer Xenium In Situ data (Janesick *et al.* 2023) and revealed differentially expressed genes within one cancer cell subtype between different histological regions (Supplementary Methods and Figs. S7 and S8, available as supplementary data at *Bioinformatics* online). In addition, we provide a tutorial on how to import previously published data of a pulmonary fibrosis TMA (Vannan *et al.* 2025)  into an InSituExperiment object and perform preprocessing steps (Fig. S9, available as supplementary data at *Bioinformatics* online).

## 3 Discussion

In this work, we introduce InSituPy, a comprehensive Python-based framework, designed to simplify the handling, analysis, and visualization of multi-sample spatial omics data. While state-of-the-art Python-based analysis frameworks such as SpatialData (Marconato *et al.* 2025) or Squidpy (Palla *et al.* 2022) focus on a sample-level analysis, InSituPy allows handling of data at both the sample and experiment levels. With functions to read, write, query and analyze the datasets, InSituPy lays the foundation for large spatial omics projects, including multiple tissue sections or tissue microarrays from large patient cohorts. For the multi-sample analysis of such datasets, InSituPy provides functionalities to conduct analysis within and across samples using a simple syntax.

On a sample level, currently published frameworks such as SpatialData structure the data based on the geometric properties of the modalities, e.g. as shapes or points. In contrast, InSituPy introduces a hierarchical data structure which groups the modalities into biologically meaningful layers, making analyses more intuitive and more accessible for non-bioinformaticians and medical experts. To allow interoperability between both frameworks, we implemented bidirectional conversion functions, giving InSituPy users access to data analysis workflows developed for SpatialData. In the future, we aim to further strengthen the support of SpatialData while also introducing advanced data formats for metadata handling such as ehrapy (Heumos *et al.* 2024).

A central step in the analysis of spatial omics data is the alignment of concurrently generated image data, which often requires laborious manual steps. Based on previously published analysis pipelines (Wirth *et al.* 2023) and similarly to R-based tools like VoltRon (Manukyan *et al.* 2023), InSituPy uses the computer vision toolbox OpenCV to facilitate an efficient and automated alignment of images from histological or immunofluorescent stainings. To verify results and develop hypotheses during analysis, interactive visualization of the data is crucial. Based on the napari framework (Sofroniew *et al.* 2024), InSituPy offers visualization of image data and cellular omics data of multiple datasets as well as the handling of histological annotations or regions, facilitating the integration of pathological expert knowledge and opening new ways of generating hypotheses and driving translational research.

All functionalities were demonstrated on a Xenium In Situ breast cancer dataset; however, InSituPy’s data structure is suitable for all types of imaging-based and sequencing-based spatial transcriptomics as well as spatial proteomics methodologies. Technology-agnostic data import strategies are available and explained in the documentation. Furthermore, InSituPy supports bidirectional conversion with SpatialData objects, enabling data import from various spatial omics technologies through the spatialdata-io ecosystem while maintaining InSituPy’s biology-inspired hierarchical structure for downstream analysis. Notably, benchmarking of InSituPy’s native Xenium reader against its spatialdata-io counterpart also highlights that specialized readers optimized for specific data formats can significantly improve loading performance.

In conclusion, InSituPy represents the first Python-based framework for multi-sample spatial omics analysis that seamlessly integrates sample-level and experiment-level data handling. Through its biology-inspired hierarchical structure and comprehensive documentation, InSituPy establishes standardized analysis workflows that improve accessibility for non-bioinformaticians and disease experts.

## Supplementary Material

btag073_Supplementary_Data

## Data Availability

Previously published data from Vannan *et al.* is deposited at GEO under accession number GSE250346. Data from Janesick et al. is available here: https://www.10xgenomics.com/products/xenium-in-situ/preview-dataset-human-breast. Download functions for easy access to demo datasets are implemented in InSituPy (see Supplementary Table S3).

## References

[btag073-B1] Badia-i-Mompel P , Vélez Santiago J, Braunger J et al decoupleR: ensemble of computational methods to infer biological activities from omics data. Bioinforma Adv 2022;2:vbac016. 10.1093/bioadv/vbac016 PMC9710656 36699385

[btag073-B2] Bankhead P , Loughrey MB, Fernández JA et al QuPath: open source software for digital pathology image analysis. Sci Rep 2017;7:16878. 29203879 10.1038/s41598-017-17204-5 PMC5715110

[btag073-B3] Black S , Phillips D, Hickey JW et al CODEX multiplexed tissue imaging with DNA-conjugated antibodies. Nat Protoc 2021;16:3802–35. 34215862 10.1038/s41596-021-00556-8 PMC8647621

[btag073-B4] Goldsborough T , O’Callaghan A, Inglis F et al A novel channel invariant architecture for the segmentation of cells and nuclei in multiplexed images using InstanSeg. 2024. 2024.09.04.611150.

[btag073-B5] Heumos L , Ehmele P, Treis T et al An open-source framework for end-to-end analysis of electronic health record data. Nat Med 2024;30:3369–80. 39266748 10.1038/s41591-024-03214-0 PMC11564094

[btag073-B6] Janesick A , Shelansky R, Gottscho AD, et al High resolution mapping of the tumor microenvironment using integrated single-cell, spatial and in situ analysis. Nat Commun 2023;14:8353. 38114474 10.1038/s41467-023-43458-x PMC10730913

[btag073-B7] Ke R , Mignardi M, Pacureanu A et al In situ sequencing for RNA analysis in preserved tissue and cells. Nat Methods 2013;10:857–60. 23852452 10.1038/nmeth.2563

[btag073-B8] Kuleshov MV , Jones MR, Rouillard AD et al Enrichr: a comprehensive gene set enrichment analysis web server 2016 update. Nucleic Acids Res 2016;44:W90–7. 27141961 10.1093/nar/gkw377 PMC4987924

[btag073-B9] Lee JH , Daugharthy ER, Scheiman J et al Highly multiplexed subcellular RNA sequencing in situ. Science 2014;343:1360–3. 24578530 10.1126/science.1250212 PMC4140943

[btag073-B10] Lowe DG. Distinctive image features from scale-invariant keypoints. Int J Comput Vis 2004;60:91–110.

[btag073-B11] Lubeck E , Coskun AF, Zhiyentayev T et al Single-cell in situ RNA profiling by sequential hybridization. Nat Methods 2014;11:360–1. 24681720 10.1038/nmeth.2892 PMC4085791

[btag073-B12] Manukyan A , Bahry E, Wyler E et al VoltRon: a spatial omics analysis platform for multi-resolution and multi-omics integration using image registration. 2023. 2023.12.15.571667.

[btag073-B13] Marconato L , Palla G, Yamauchi KA et al SpatialData: an open and universal data framework for spatial omics. Nat Methods 2025;22:58–62. 38509327 10.1038/s41592-024-02212-x PMC11725494

[btag073-B14] Martinelli AL , Rapsomaniki MA. ATHENA: analysis of tumor heterogeneity from spatial omics measurements. Bioinformatics 2022;38:3151–3. 35485743 10.1093/bioinformatics/btac303 PMC9154280

[btag073-B15] Muja M , Lowe DG. Fast Approximate Nearest Neighbors with Automatic Algorithm Configuration. In: *Proceedings of the Fourth International Conference on Computer Vision Theory and Applications.* Lisboa, Portugal: SciTePress - Science and Technology Publications, 2009, 331–340.

[btag073-B16] Palla G , Spitzer H, Klein M et al Squidpy: a scalable framework for spatial omics analysis. Nat Methods 2022;19:171–8. 35102346 10.1038/s41592-021-01358-2 PMC8828470

[btag073-B17] Peters Couto BZ , Robertson N, Patrick E et al MoleculeExperiment enables consistent infrastructure for molecule-resolved spatial omics data in bioconductor. Bioinformatics 2023;39:btad550. 37698995 10.1093/bioinformatics/btad550 PMC10504467

[btag073-B18] Raudvere U , Kolberg L, Kuzmin I et al g: profiler: a web server for functional enrichment analysis and conversions of gene lists (2019 update). Nucleic Acids Res 2019;47:W191–8. 31066453 10.1093/nar/gkz369 PMC6602461

[btag073-B19] Righelli D , Weber LM, Crowell HL et al SpatialExperiment: infrastructure for spatially resolved transcriptomics data in R using bioconductor. Boeva V (ed.). Bioinformatics 2022;38:3128–31. 35482478 10.1093/bioinformatics/btac299 PMC9154247

[btag073-B20] Rodriques SG , Stickels RR, Goeva A et al Slide-seq: a scalable technology for measuring genome-wide expression at high spatial resolution. Science 2019;363:1463–7. 30923225 10.1126/science.aaw1219 PMC6927209

[btag073-B21] Schürch CM , Bhate SS, Barlow GL et al Coordinated cellular neighborhoods orchestrate antitumoral immunity at the colorectal cancer invasive front. Cell 2020;182:1341–59.e19. 32763154 10.1016/j.cell.2020.07.005 PMC7479520

[btag073-B22] Sofroniew N , Lambert T, Bokota G et al napari: a multi-dimensional image viewer for Python. Zenodo, 2024. 10.5281/zenodo.13863809

[btag073-B23] Ståhl PL , Salmén F, Vickovic S et al Visualization and analysis of gene expression in tissue sections by spatial transcriptomics. Science 2016;353:78–82. 27365449 10.1126/science.aaf2403

[btag073-B24] Szklarczyk D , Gable AL, Lyon D et al STRING v11: protein–protein association networks with increased coverage, supporting functional discovery in genome-wide experimental datasets. Nucleic Acids Res 2019; 47: D607–D613. 30476243 10.1093/nar/gky1131 PMC6323986

[btag073-B25] Vannan A , Lyu R, Williams AL et al Spatial transcriptomics identifies molecular niche dysregulation associated with distal lung remodeling in pulmonary fibrosis. Nat Genet 2025;57:647–58. 39901013 10.1038/s41588-025-02080-x PMC11906353

[btag073-B26] Virshup I , Bredikhin D, Heumos L, et al The scverse project provides a computational ecosystem for single-cell omics data analysis. Nat Biotechnol 2023;41:604–6. 37037904 10.1038/s41587-023-01733-8

[btag073-B27] Virshup I , Rybakov S, Theis FJ et al anndata: Annotated data*bioRxiv* 2021. 2021.12.16.473007.

[btag073-B28] Wirth J , Huber N, Yin K et al Spatial transcriptomics using multiplexed deterministic barcoding in tissue. Nat Commun 2023;14:1523. 36934108 10.1038/s41467-023-37111-w PMC10024691

[btag073-B29] Xia C , Fan J, Emanuel G et al Spatial transcriptome profiling by MERFISH reveals subcellular RNA compartmentalization and cell cycle-dependent gene expression. Proc Natl Acad Sci U S A 2019;116:19490–9. 31501331 10.1073/pnas.1912459116 PMC6765259

