# Supplementary material for: InSituPy: a framework for histology-guided, multi-sample analysis of single-cell spatial omics data
Source: Bioinformatics. 2026 Feb 15;42(3):btag073. doi: 10.1093/bioinformatics/btag073 (PMC12988772; doi:10.1093/bioinformatics/btag073)
Supplement: btag073_Supplementary_Data [file btag073_supplementary_data.zip › InSituPy_manuscript_WirthJ_Bioinformatics_SuppData_Final.pdf]

Supplementary Data

InSituPy: a framework for histology-guided, multi-sample analysis of single-cell spatial omics data

Johannes Wirth[1]*, Anna Chernysheva[1], Birthe Lemke[1], Isabel Giray[1], Katja Steiger[1,2]*

[1] Institute of Pathology, School of Medicine and Health, Technical University Munich, Munich, Germany

[2] German Cancer Consortium (DKTK) partner site Munich, Munich, Germany

* To whom correspondence should be addressed. E-mail: j.wirth@tum.de, katja.steiger@tum.de

Table of Contents

Supplementary Methods 2

- Package architecture 2
- Data analysis 4
- Demonstration of data analysis with InSituPy using a Xenium breast cancer dataset 6

Supplementary Figures 7

Supplementary Tables 15

References 16

Supplementary Methods

Package architecture

Implementation of InSituPy

The InSituPy framework is built in Python >=v3.10 and offers a multi-sample and sample-level analysis of single-cell spatial omics data. All code is licensed under the "BSD 3-Clause" and available on Github (https://github.com/SpatialPathology/InSituPy). The software is archived on Zenodo (DOI: 10.5281/zenodo.18459471) with the version used for this publication (v0.11.0b3) available at DOI: 10.5281/zenodo.18459472. The package can be also installed via PyPi using the command *pip install insitupy-spatial*. Information about installation and usage as well as tutorials are available on Github and additional documentation is built using Sphinx and hosted on Read the Docs. InSituPy has been tested on Windows 11, Linux (SLES 15 SP6), and macOS (Sequoia 15.5, Apple Silicon M3).

InSituPy dependencies

The InSituPy framework depends on following packages: *scanpy*, *loompy* (single-cell omics data analysis); *dask*, *opencv*, *zarr* (image data operations); *geopandas*, *rasterio* (spatial data operations); *napari* (visualization); *numpy*, *scikit*, *scipy* (general mathematical operations); *matplotlib*, *seaborn*, *adjusttext* (plotting); *pandas*, *toml, fastparquet, pyarrow* (handling of general data formats), *pytest* (code testing).

Architecture of InSituData object

To achieve optimal storage and handling of the different data modalities (images, cellular transcriptomes, cellular boundaries, transcripts, geometric data), they are structured into biologically meaningful entities using specialized data classes and collected within an *InSituData* object. Loading of a certain modality can be invoked using the respective '*load_{modality}*' function. A summary of all used data formats and packages can be found in **Table S2**. The different data classes are described subsequently.

Image data

Image data is handled using the *ImageData* class, allowing the addition of multiple images and associated metadata such as resolution of the image or other OME metadata. To efficiently load large image data, InSituPy exploits the resource-saving capabilities of the *dask* framework and stores the data in the *Zarr* format allowing lazy loading of the data. For export to open source tools like QuPath images can be saved as OME-TIFF. The image resolution is pivotal because all data classes of InSituPy use metric units (usually µm) as basis for building a common coordinate system. Images can be saved either as *zarr*-formatted image file (Miles *et al.* 2020) or alternatively as *OME-TIFF* (Besson *et al.* 2019) to allow compatibility with bioimage analysis software such as QuPath (Bankhead *et al.* 2017).

Cellular data

At the center of the analysis is the cellular data which is stored in a *CellData* object and consists of the single-cell omics data and the cellular boundaries of each cell. Multiple CellData objects can be summarized in layers of a MultiCellData object. This allows the integration of results from multiple alternative cell segmentation runs within an InSituData object. During the analysis specific layers can be selected using the *cells_layer* argument. Within one CellData object the single-cell omics data is stored as *AnnData* (Virshup *et al.* 2021) object which allows compatibility with *scverse* analysis packages, including *SpatialData* (Marconato *et al.* 2025), *Squidpy* (Palla *et al.* 2022) or *Scanpy* (Wolf, Angerer, and Theis 2018). The information about cellular boundaries is stored in a *BoundariesData* object as pyramidal dask arrays allowing fast visualization in the form of a labels layer in *napari*. Information about the transcript locations and identities, as measured in in situ sequencing or in situ hybridization methods, is stored as *dask* (Rocklin 2015) data frame in the ".transcripts" attribute.

Omics data of spatial units

Spatial transcriptomics data from spot-based technologies like Visium or from multi-cellular anatomical structures like functional tissue units is stored in a SpatialUnitsData object. This object contains the geometric shapes stored as shapely (Gillies, and others 2007) objects inside a GeoPandas (Jordahl 2014) dataframe and the spatial omics data as AnnData (Virshup *et al.* 2021) object, maintaining compatibility with scverse (Virshup *et al.* 2023) analysis packages including SpatialData (Marconato *et al.* 2025), Squidpy (Palla *et al.* 2022), and ScanPy (Wolf, Angerer, and Theis 2018).

Histological annotations

The complexity of histological annotations is handled using the *ShapesData* object. Based on a unique *key* as reference (e.g. name of the pathologist or overarching type of the annotation) and a *class* (e.g. “tumor”, “immune cells”), ShapesData allows the addition of geometric information. The geometric shapes are stored as shapely (Gillies, and others 2007) object inside a GeoPandas (Jordahl 2014) dataframe, which facilitates the application of advanced functions from both packages. Geometric data is saved as GeoJSON file. Within geometric annotations, InSituPy differentiates between “annotations” and “regions” (**Figure 1 D**). The main difference between the two types is that an annotation is allowed to have multiple geometric objects per object name while a region is required to provide a unique name for each geometry. Further, annotations can consist of any kind of geometric shapes (e.g. polygons, lines or points), while regions can only consist of polygons. Annotations are meant to reflect histological annotations (e.g. tumor, necrosis) where multiple geometries with the same name are expected, while regions are meant to reflect regions such as TMA cores where each geometry possesses a unique name. These two possibilities are reflected by two daughter classes of *ShapesData*: *AnnotationsData* and *RegionsData*.

Architecture of InSituExperiment object

To allow a comprehensive analysis of datasets with multiple samples, various *InSituData* objects can be combined in an *InSituExperiment* object, connecting the data with corresponding metadata (e.g. clinical data or experimental data). Different strategies are implemented to build an InSituExperiment object (Supp. Figure 1 A), including (i) the direct generation from an InSituData object based on regions, (ii) the addition of individual InSituData objects to an empty or existing InSituExperiment object or (iii) directly from a configuration file. Metadata can either be added using the *append_metadata* function or specified in the configuration file. An InSituExperiment dataset can be assembled using different strategies (**Figure 1 E**): (i) histological regions can be used to generate it, e.g. if one dataset contains multiple tissue sections or TMA cores, each originating from different samples. (ii) Individual InSituData object can be added manually to an existing InSituExperiment. Afterwards, sample-specific metadata can be assigned to the data. And (iii), an InSituExperiment can also be created based on a configuration file containing the data directories and the corresponding metadata.

Reading and writing of data

Both on a multi-sample and single-sample level functions are implemented to save the *InSituExperiment* or *InSituData* object to disk. After saving, the data can be read using either *InSituExperiment.read()* or *InSituData.read().* Modalities can be loaded using the respective loading functions. Image data and transcript data are loaded lazily. To read data from different single-cell spatial omics technologies, distinct reading functions are implemented (e.g. *read_xenium* for the Xenium In Situ method or *read_qupath* and *read_qupath_project* for data exported from QuPath). Further, documentation on how to read custom data is available online. Data can be saved using either the functions `saveas` (saves both static and variable data) or `save` (saves only the variable data). These functions are available for both InSituData and InSituExperiment. For performance tests shown in **Figure S2**, a Lenovo ThinkPad T16 Gen 1 laptop equipped with an AMD Ryzen 7 PRO 6850U processor (8 cores, 16 logical processors), 32 GB RAM, and running Microsoft Windows 11 Education (Build 26100) was used.

Data analysis

Download of demo datasets

To facilitate a fast implementation and testing of InSituPy, functions to download multiple different demo datasets of the Xenium In Situ technology are implemented. This includes Xenium v1 datasets from different human samples: breast cancer, non-diseased kidney, pancreatic cancer, skin melanoma, brain cancer, lung cancer and lymph node. Further, a lymph node sample from the Xenium 5K technology and a test dataset with small data size can be downloaded through these functions. The URLs used for the download can be found in **Table S3**.

Preprocessing functions

An example preprocessing workflow for Xenium In Situ data is provided in the documentation. Important *Scanpy*-based (Wolf, Angerer, and Theis 2018) preprocessing steps such as normalization, transformation and dimensionality reduction were summarized in the functions *normalize_and_transform* and *reduce_dimensions*, respectively. For normalization, the function uses Scanpy's *normalize_total* function and for transformation both log-transformation and square-root transformation are implemented.

Automated image registration of histological and immunofluorescent stainings

For the automated image registration pipeline, functions from the computer vision library OpenCV (*v4.8.0.76*) were used. This pipeline aligns the subsequently stained images to the nuclear image acquired during the spatial omics measurement. Before the registration, both images were downscaled to a maximum width of 4000 pixels to reduce the memory consumption. *Scale-Invariant Feature Transform* (Lowe 2004) was used with default parameters to identify keypoints in both images. Subsequently, the *Fast Library for Approximate Nearest Neighbors* (FLANN)(Muja and Lowe 2009) was used to match the keypoints in both images using the parameters *kdtree=5* and *checks=50*. Lowe's ratio test (Lowe 2004) was used to eliminate false matches using a ratio threshold of *0.7*. If not enough matched keypoints were found, the algorithm automatically tested whether flipping the image vertically or horizontally led to better results and continues with the best of these options. Before calculation of the transformation matrix, the remaining keypoints were rescaled to the size of the original input images. For a perspective transformation, the *findHomography* function and for an affine transformation the *estimateAffine2D* function were used. In both functions, the most robust matches were selected using *random sample consensus algorithm*, followed by the calculation of an affine or perspective transformation matrix. This transformation matrix was then used to transform the histological image using the functions *warpPerspective* or *warpAffine*, respectively. A demonstration of the automated registration pipeline can be found in the documentation.

Histological annotation in QuPath and import into InSituPy

For annotation of histological images in external software, the open-source software *QuPath* (Bankhead *et al.* 2017) was tested and workflows for its use were implemented. The implementation of the workflows was done in version *0.5.1* of QuPath. Annotations can be applied using QuPath's standard annotation tools and exported using *File > Export objects as GeoJSON*. To import the geometric shapes as either annotations or regions two import functions (*import_annotations* and *import_regions*) are provided. Since InSituPy uses metric units, it is important to provide a scale factor in µm per pixel. The scale factor corresponds to the resolution of the image on which the annotations were generated. To determine which cells are located within a certain annotation, the function *assign_annotations* can be used. It utilizes mainly functions from the *shapely* (v2.0.7)(Gillies, and others 2007) and *GeoPandas* packages (v1.0.1)(Jordahl 2014). Examples of this workflow are provided in the documentation. All annotations were performed by a pathologist.

Interactive visualization using napari

For interactive visualization of the single-cell spatial omics data a viewer was implemented using napari (Sofroniew *et al.* 2024) (*v0.5.6*). The viewer can be invoked from a *InSituData* or *InSituExperiment* object using the *show* function. In case of an InSituExperiment object the index of the respective dataset needs to be provided. Images are added lazily to the viewer using the *Dask* framework (*v2025.3.0*)(Rocklin 2015) from either a *zarr*-formatted (Miles *et al.* 2020) or *OME-TIFF* (Besson *et al.* 2019) formatted image file. To visualize and interact with the data, different widgets were implemented (**Figure S5** and **6**). Cellular data can be displayed as points layer using the "Show data" widget and filtered using "Filter cells" widgets. Filtering can be used e.g. to display only a certain cell type. Cellular boundaries can be visualized as labels layer using the "Show boundaries" widget. To localize and highlight a certain cell, the "Navigate to cell" widget can be used. Annotations or regions can be added using the "Add geometries" widget. For geometric annotations or regions, a shapes layer is added while for point annotations a points layer is added to the viewer. Already existing annotations or regions can be displayed using the "Show geometries" widget. Interactive

visualization features using *napari* require a graphical display environment and may not be available on headless systems such as remote servers without X11 forwarding or display configuration. For such environments, InSituPy provides alternative static visualization functions using *matplotlib*.

Differential gene expression analysis

Differential gene expression analysis is based on the *rank_genes_groups* function from Scanpy (v1.10.3). The analysis can be performed either on the sample level using the function *differential_gene_expression* or on the experiment level using the class function *dge* of an *InSituExperiment* object. Further, the analysis can be limited to annotations, regions or observation categories (e.g. cell types).

GO term enrichment analysis

InSituPy provides functionalities to connect to APIs from different GO term enrichment analysis web servers including STRING (Szklarczyk *et al.* 2019), g:profiler (Raudvere *et al.* 2019), and Enrichr (Kuleshov *et al.* 2016). Packages used to connect to the APIs include *requests* (v2.32.3), *gprofiler-official* (v1.0.0) and *gseapy* (v1.1.8). The exact statistical tests and calculation of the false discovery rate (FDR) depend on the web server used and details can be found in the documentation of the respective publications. Enrichment scores are returned as -log10(FDR). The gene ratio was calculated as fraction of genes of a specific pathway that were present in the query list.

Distance-dependent gene expression analysis

The function *calc_distance_of_cells_from* uses the *geopandas* package to calculate the Euclidean distance between each cell to the closest point on a specified annotation or region. If, in case of annotations, multiple annotation objects exist only the closest annotation is used as reference.

Cellular density analysis

For cellular density analysis two different strategies are implemented to calculate the density of a specified cell type: (i) Kernel-density estimation using Gaussian kernels as implemented in *scipy* (v1.15.2), and (ii) log density estimation as implemented in *mellon* (Otto *et al.* 2024) (v1.6.1). The density results of both methods can be chosen to be clipped to their 5th and 95th percentile as recommend in ref. (Otto *et al.* 2024).

Plotting functions

InSituPy contains different plotting functionalities to visualize the results of above-mentioned analyses. This includes volcano plots, pie plots, dot plots, bar plots and spatial scatter plots which are plotted using functionalities from the packages *matplotlib* (v3.10.1), seaborn (v0.13.2), and *adjustText* (v1.3.0).

Fluorescence Signal Quantification

Fluorescence signal intensity can be quantified for individual cells or nuclei using the *quantify_signal* function. For each cellular compartment, one can either compute the mean or median fluorescence intensity across all pixels within the corresponding segmentation mask. For large images, a tiled processing approach is employed to manage memory constraints. In brief, images and masks are divided into overlapping tiles to ensure continuous coverage at tile boundaries. Quantification is performed independently on each tile, and measurements from overlapping regions are aggregated by retaining a single measurement per cell across all tiles. Optional downsampling can be applied to reduce computational burden while preserving spatial signal distributions. Cell identities are mapped from segmentation mask values to corresponding cell identifiers, and quantification results are stored as cell-level observations for downstream analysis.

Spatial Alignment of Multi-Modal Data

To integrate spatial transcriptomics data from different modalities (e.g., Visium spots onto Xenium cells), we perform spatial alignment using pre-computed transformation matrices. Transformation matrices can be computed from external tools (e.g. Xenium Explorer or Fiji's *BigWarp*). The alignment function takes spatial units from a source dataset (here the Visium spots) and applies an affine transformation to register them to a reference coordinate system (here the Xenium dataset). Pixel size conversion is handled automatically by inferring resolution metadata from the respective imaging modalities, or by explicit specification of source and reference pixel sizes (in µm/pixel). Optionally, images associated with the source dataset are also transformed and transferred to the reference dataset using the same transformation parameters. This ensures geometric consistency between all aligned spatial modalities. For demonstration of this functionality data from Janesick et al.(Janesick *et al.* 2023) was used. A tutorial is available in the documentation.

Benchmarking of Xenium In Situ readers

For benchmarking, the Xenium In Situ readers of spatialdata-io (v0.5.1; paired with SpatialData v0.6.1) and InSituPy (v0.11.0-beta) were used. A detailed list of the used datasets with corresponding URLs can be found in **Table S4**. Loading time and memory consumption were tracked with the *time* or the *tracemalloc* modules, respectively.

Demonstration of data analysis with InSituPy using a Xenium breast cancer dataset

To exemplify data analysis steps in InSituPy, we used a published Xenium In Situ dataset of a breast cancer sample (Janesick *et al.* 2023). The described analysis presents a basic analysis workflow which can be also applied to other data. All code of the analysis is available in the documentation.

Identification of breast cancer subtypes using InSituPy

Cell type annotation based on marker genes revealed normal breast cells such as glandular cells and myoepithelial cells, five different subtypes of breast cancer cells as well as other cell types including immune cells and stromal cells (**Figure S7 A**). InSituPy provides visualization functionalities to explore the spatial distribution of cell types in the sample as well as the expression of genes of interest (**Figure S7 B**). Mapping all cell types on the whole specimen revealed a distinct spatial clustering of the different breast cancer subtypes (**Figure S7 C**). Annotation of the H&E image by a pathologist revealed one larger area with invasive tumor and multiple smaller areas with *ductal carcinoma in situ* (DCIS). The DCIS regions were further divided into classical DCIS, DCIS with stromal reaction and an intermediate DCIS type. These annotations correspond well with the annotations of DCIS #1 and DCIS #2 in the original publication (Janesick *et al.* 2023). Using InSituPy functionalities to investigate the cellular composition of the different annotations and regions, revealed a clear correspondence of the detected breast cancer subtypes with the pathological annotations (**Figure S7 D**). The classical DCIS matched well with breast cancer subtype 4 while DCIS with stromal reaction largely consisted of subtype 5. The intermediate typed DCIS predominantly consisted of breast cancer subtype 2 and the invasive tumor of subtypes 1 and 3, demonstrating the value of InSituPy's functions to interpret cellular subtypes based on pathological annotations.

Histology-guided differential gene expression analysis across tissue regions

A central task in omics data analysis is the detection of differentially expressed genes (DEGs). Using the structure of the InSituPy framework described above (**Figure 1 B**), the analysis can be limited to selected annotations or cell types and results can be visualized in volcano plots (**Figure 1 G**). Here, we demonstrated this functionality by investigating gene expression differences between cells of breast cancer subtype 4, corresponding to classical DCIS and cells of subtype 1, corresponding to invasive tumor cells. The analysis was limited to the respective pathological annotations and revealed 30 up- and 37 downregulated genes (**Figure S7 E**). The expression of two example genes, *KLF5* and *SERPINA3*, in a representative region is shown in **Figure S7 F**. Next, we used the DGE analysis functionalities of InSituPy to compare the gene expression of one cell type, breast cancer subtype 5, between region 2 and region 3. The analysis was restricted to cells within the pathological annotations "DCIS with stromal reaction" and resulted in 21 up- and 49-downregulated genes (**Figure S7 G**). Spatially resolved gene expression of two of the DEGs, AQP3 and SERPINA3, is shown in **Figure S7 H**, revealing clear gene expression differences in cells of breast cancer subtype 5 which could indicate further subtypes or cell states within it and underline the importance of the integration of pathological information into the analysis.

Exploration of distance-dependent changes

The distance to neighboring cells or anatomical structures as well as the density of cells influences the phenotype of cells. Exploring such distance-dependent changes can provide information about pathological processes. Utilizing either kernel density estimation or the *mellon* package for cell-state density estimation in single-cell data (Otto *et al.* 2024), InSituPy calculates the density of particular cell types within the samples, e.g. breast cancer cells or T cells (**Figure S8 A**). Further, InSituPy provides streamlined workflows to simplify distance-dependent analyses, including functions to calculate cell-cell distance relationships within the sample, as exemplified in **Figure S8 B** using the tumor centers as reference points. Subsequently, the cellular composition of the tumor can be visualized as a function of the distance to the tumor center, revealing distance-dependent changes in the tumor microenvironment (**Figure S8 C**). Further, InSituPy allows the exploration of distance-dependent expression changes of selected genes, as exemplified here for *ACTA2*, *LUM*, *MMP2*, and *CXCL12* (**Figure S8 D**).

Supplementary Figures

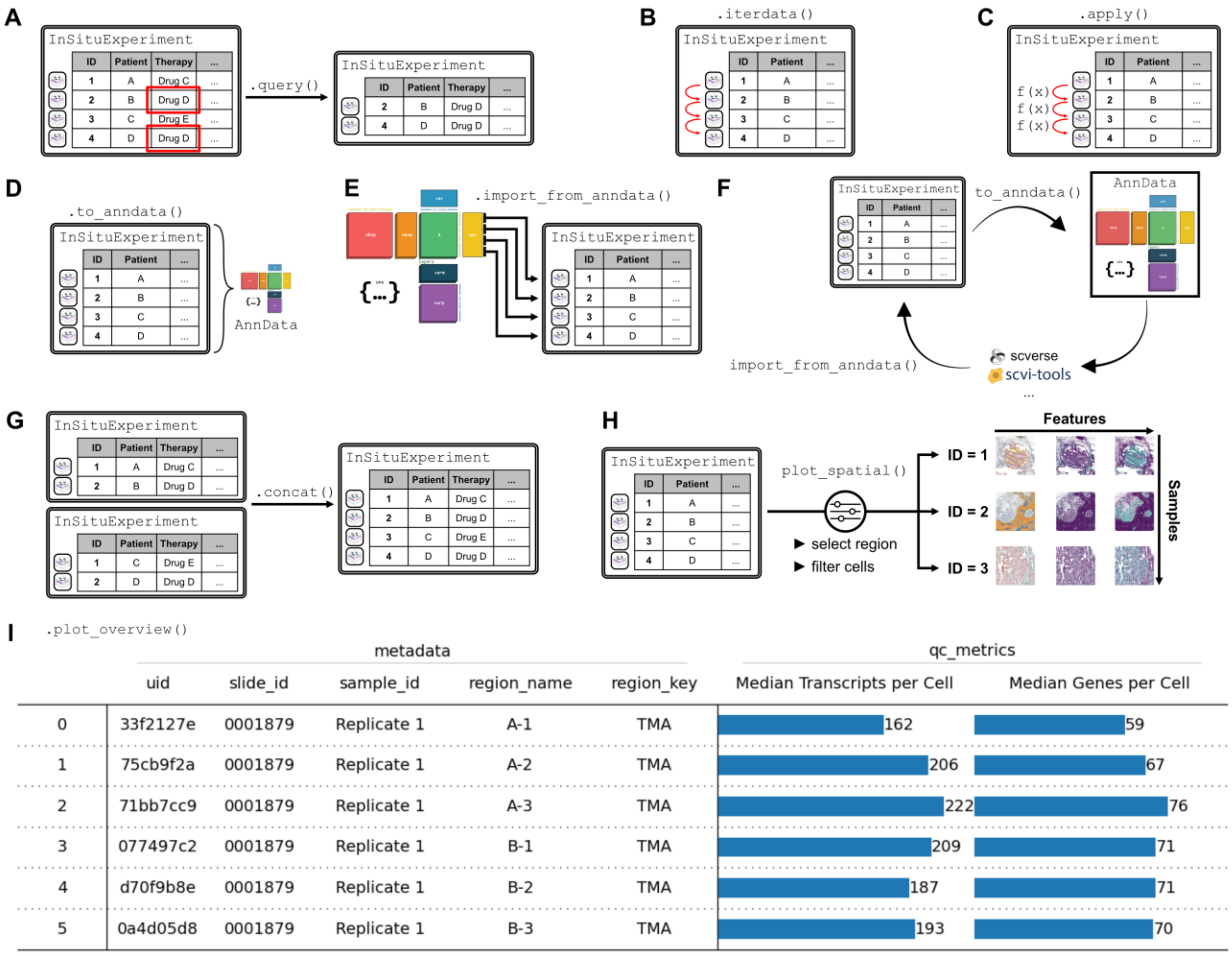

Figure S1. Experiment-level operations in InSituPy. (A-I) Schematics illustrating different functions to perform multi-sample operations in InSituPy.

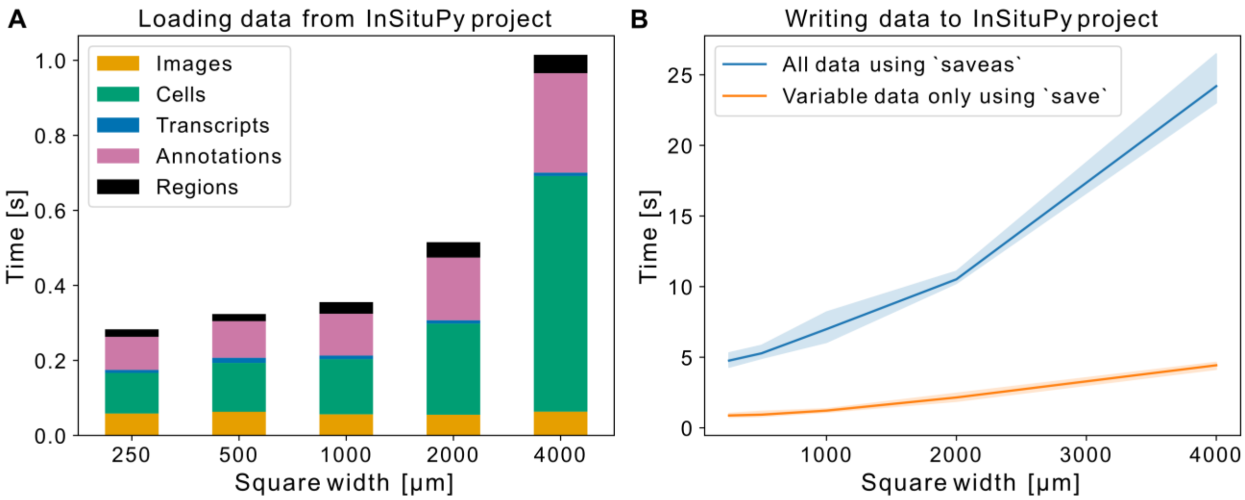

Figure S2. Loading and writing data in InSituPy. (A) Loading performance for datasets of different sizes. **(B)** Writing performance for datasets of different sizes. Writing performance was tested using the `saveas` function, saving both static and variable data, and the `save` function saving only the variable data. For the performance tests, a Lenovo ThinkPad T16 Gen 1 laptop equipped with an AMD Ryzen 7 PRO 6850U processor (8 cores, 16 logical processors), 32 GB RAM, and running Microsoft Windows 11 Education (Build 26100) was used.

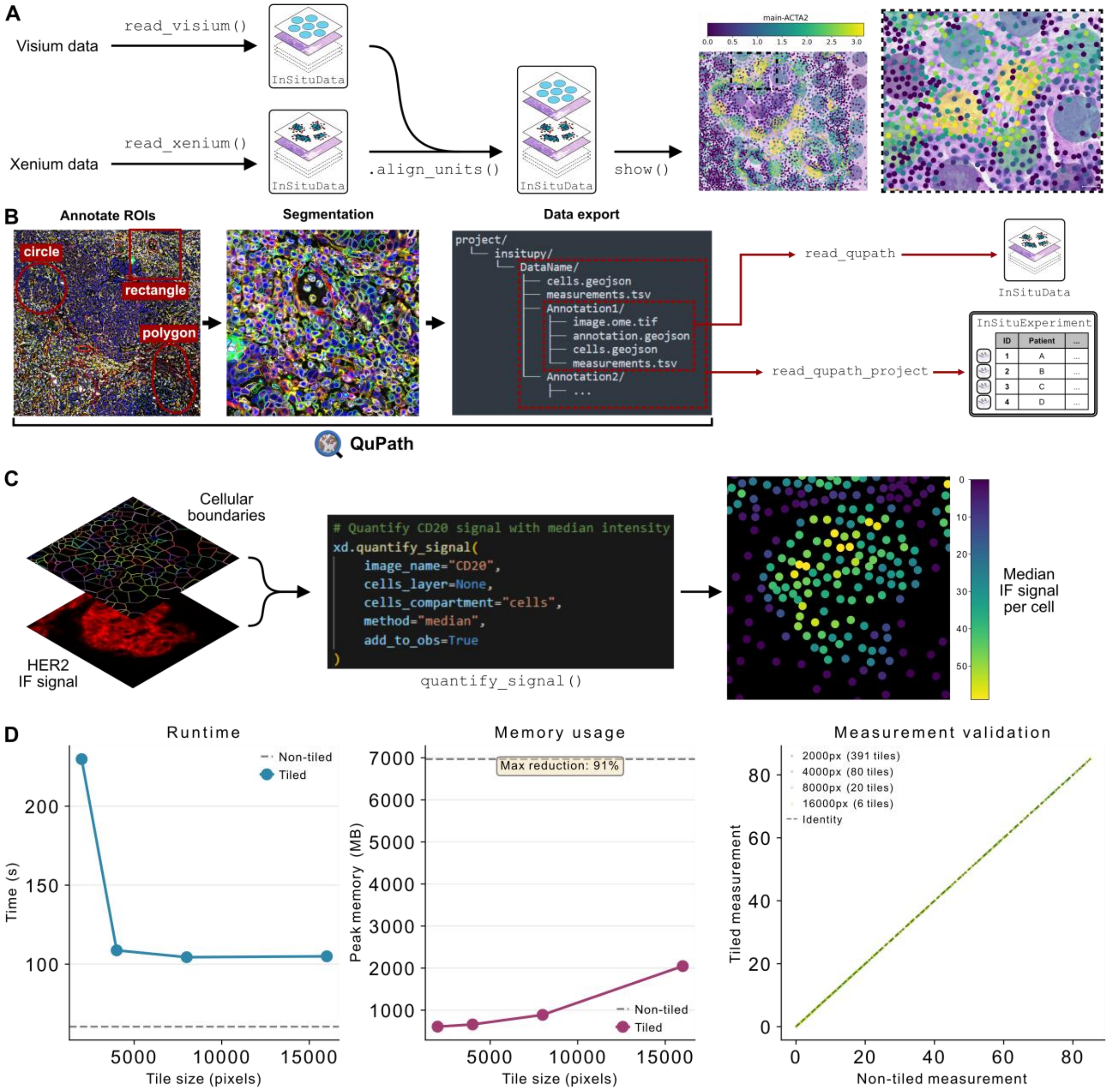

Figure S3. Multi-modal functionalities in InSituPy. (A) Workflow to integrate and visualize spot-based data (e.g. Visium) and single-cell spatial transcriptomic data (e.g. Xenium). **(B)** Overview of a workflow to preprocess multiplex immunofluorescence data in QuPath, export the data and import either individual datasets or whole data projects into InSituPy. Scripts to export the data can be found in https://github.com/SpatialPathology/InSituPy-QuPath. **(C)** Workflow to quantify signal from aligned IF images within cellular boundaries using the *quantify_signal* function. **(D)** Benchmarking of the memory-optimized, tiled version of *quantify_signal*. Benchmarking shows an increase in runtime and a decrease in memory usage when using a tiled approach. The results of the measurements are identical for both approaches.

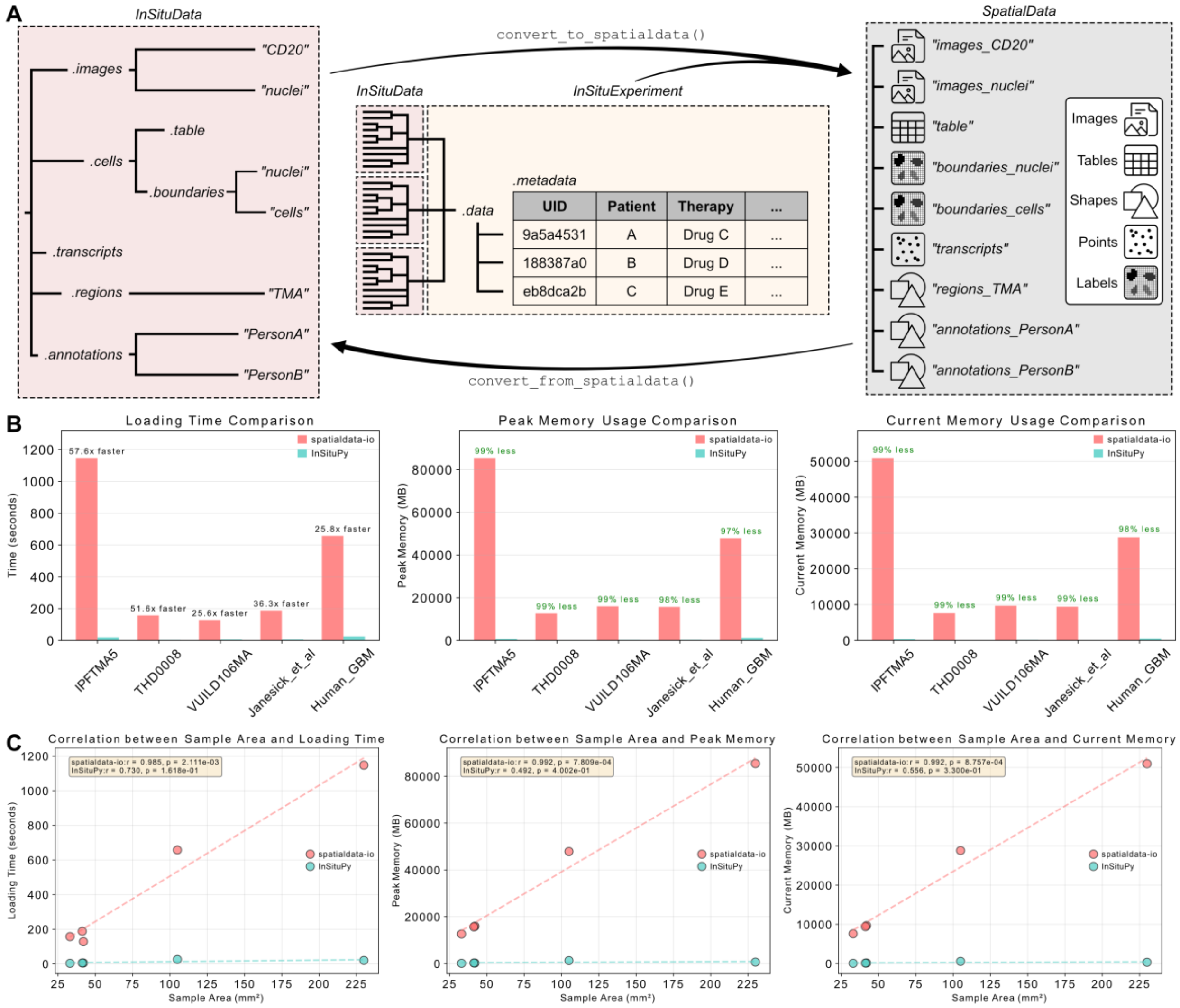

Figure S4. Interoperability with SpatialData and benchmarking against spatialdata-io. (A) Schematic showing the available conversion workflows between InSituPy and SpatialData as well as a comparison between the hierarchical, biology-focused data structure of InSituPy and the flat, geometry-focused data structure of SpatialData. **(B)** Bar plot showing benchmarking results comparing reading different Xenium In Situ datasets using either the spatialdata-io xenium reader or the InSituPy read_xenium function. **(C)** Correlation of benchmarking metrics with area of the sample.

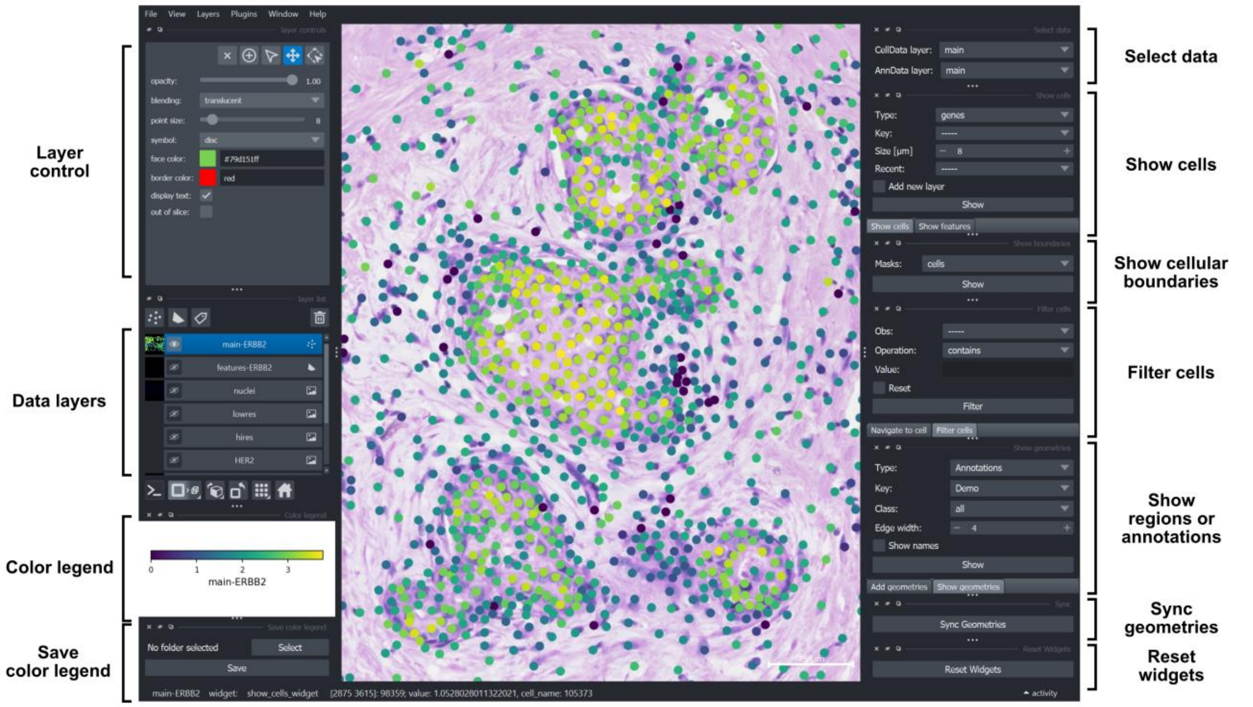

Figure S5. Data visualization in napari viewer. Screenshot showing the interactive napari viewer integrated in InSituPy and short description of the available widgets.

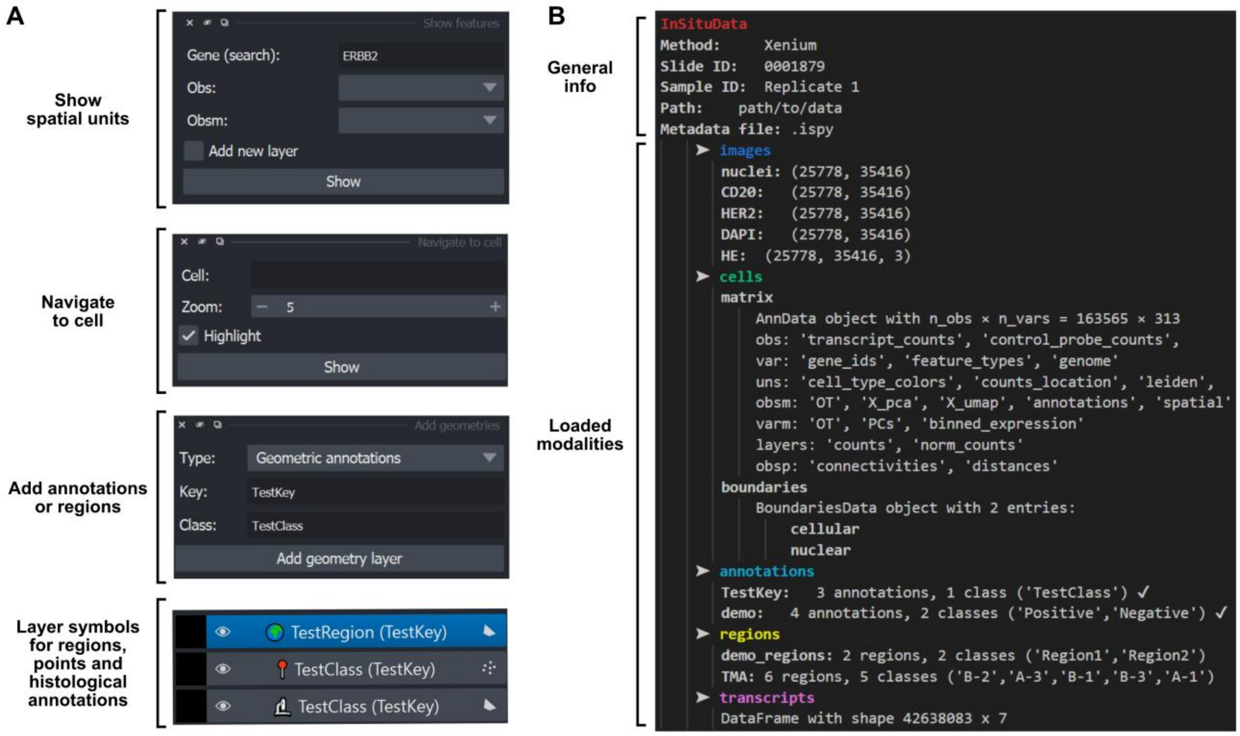

Figure S6. Napari viewer widgets and data display in InSituPy. (A) Screenshots of additional widgets in napari viewer. **(B)** String representation of InSituData object.

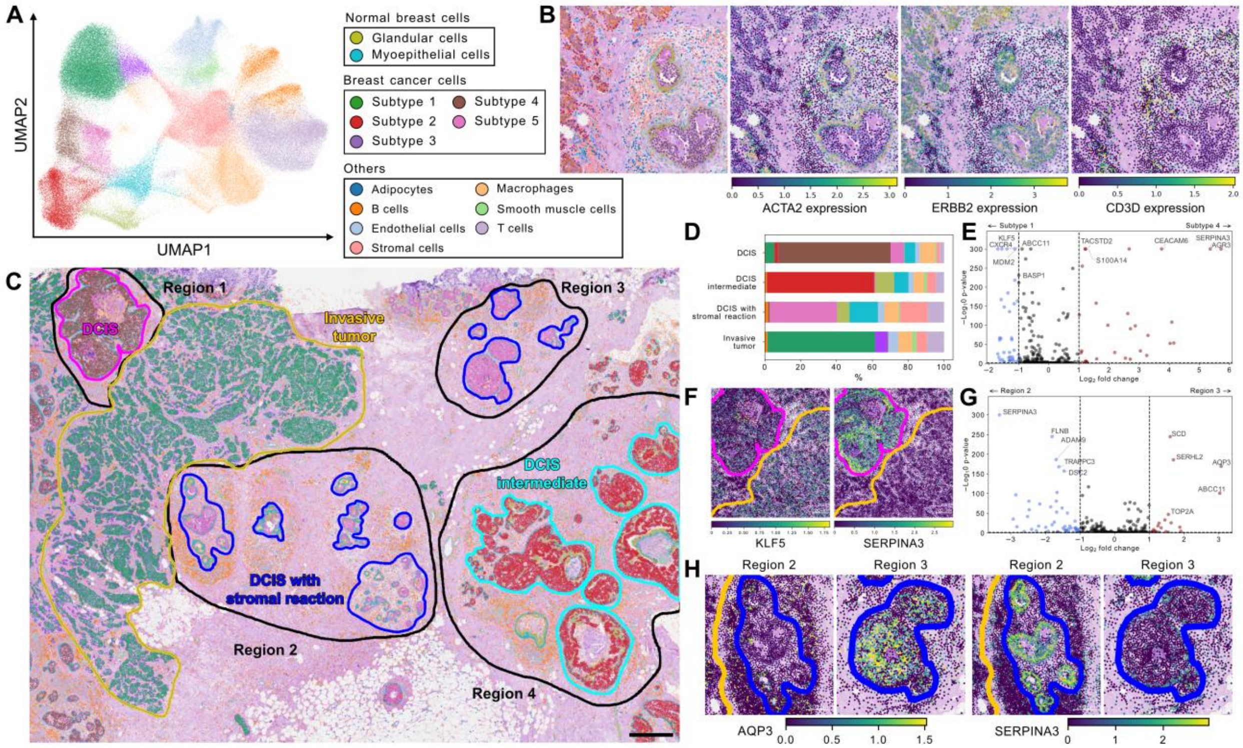

Figure S7. Histology-guided transcriptomic analyses using InSituPy. (A) Two-dimensional UMAP embedding of an example breast cancer *Xenium In Situ* dataset. Colors denote the cell type annotation. **(B)** Overlay of transcriptomic data and an image of the H&E stained tissue section. Colors denote the cell type or the gene expression of *ACTA2*, *ERBB2* or *CD3D*, respectively. **(C)** Overlay of cells depicted as points and pathological annotations and regions on an H&E stained breast cancer section. Colors of the points denote the cell type. Scale bar: 500 µm. **(D)** Bar chart showing the results of the cellular composition analysis in the pathological annotations depicted in C. Colors correspond to cell types shown in B. **(E)** Volcano plot showing the differentially expressed genes in breast cancer subtype 4 within the "DCIS" annotation compared to subtype 1 within the "Invasive" annotation. **(F)** Cellular gene expression of *KLF5* and *SERPINA3* in an example region including both DCIS and invasive tumor. Annotation colors correspond to D. **(G)** Volcano plot showing the differentially expressed genes in cells of breast cancer subtype 5 in DCIS with stromal reaction in region 3 compared to region 2. **(H)** Spatially resolved cellular gene expression of *AQP3* and *SERPINA3* in region 3 and 2 respectively. Annotation colors correspond to D. UMAP: Uniform Manifold Approximation and Projection; H&E: Hematoxylin and eosin.

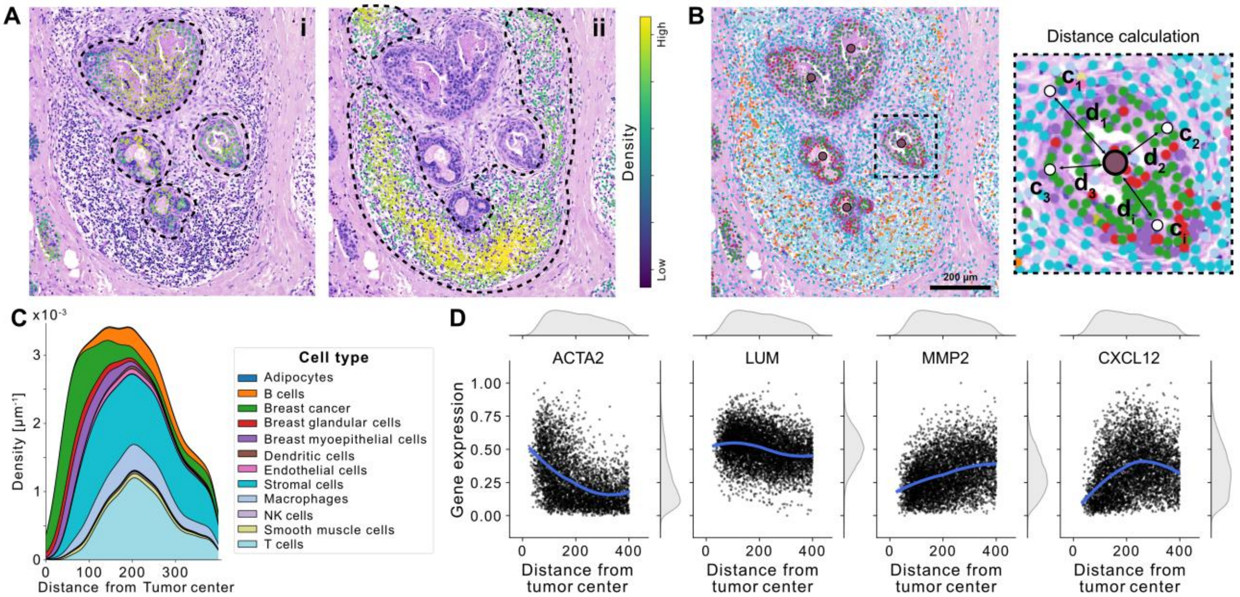

Figure S8. Cell type density and distance-dependent gene expression changes. (A) Overlay of cellular data as points and H&E stained tissue section of breast cancer example dataset. Colors denote the density of breast cancer cells (i) and T cells (ii). Density was calculated using the mellon (Otto *et al.* 2024) package. **(B)** Schematic illustrating the distance calculation and annotations. In this example, a point annotation of the tumor center was used as an example. **(C)** Cellular composition with increasing distance from the tumor center. Colors denote the cell types. **(D)** Scatter plot showing the gene expression of different genes with increasing distance from the tumor center. Each dot represents the gene expression in one cell. The smoothed line was calculated using LOESS regression. LOESS: Locally estimated scatterplot smoothing.

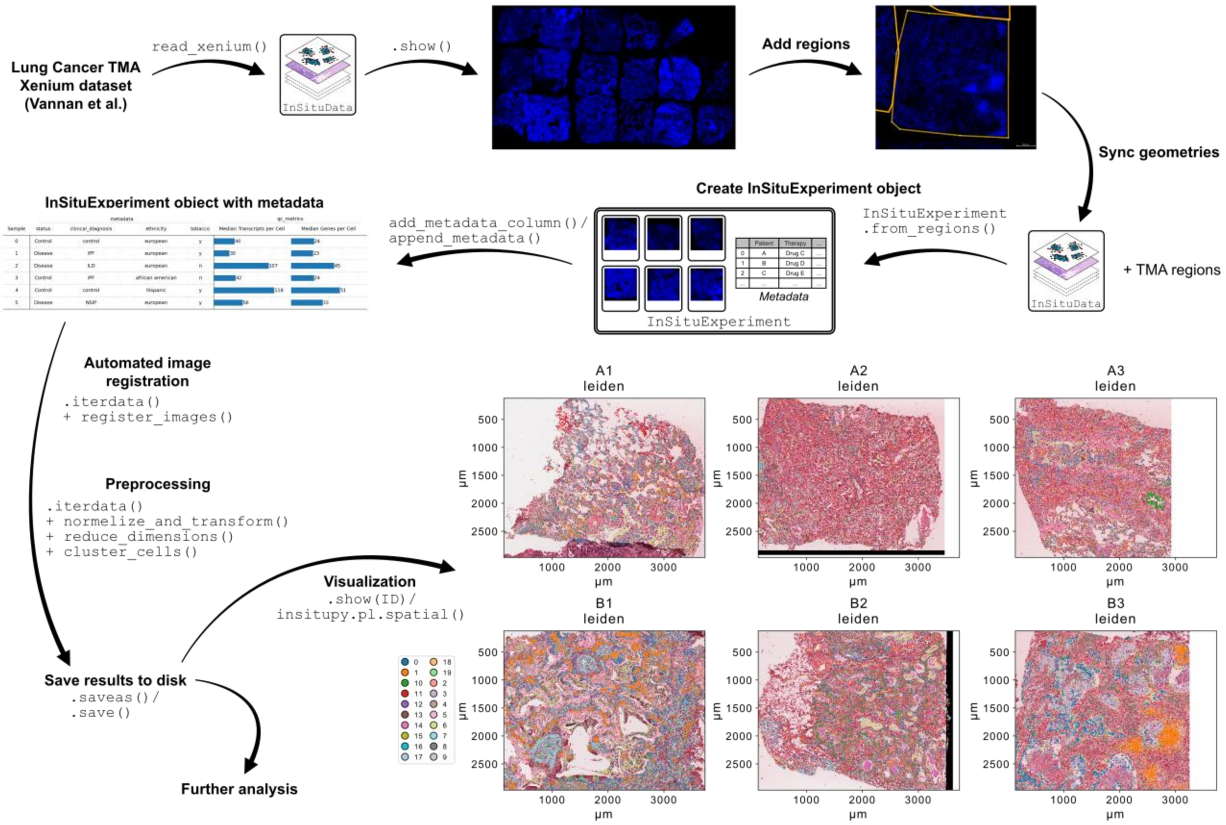

Figure S9. Demonstration how to import multi-sample datasets into InSituPy. For demonstration, a published lung cancer TMA Xenium In Situ dataset from Vannan et al.(Vannan *et al.* 2025) was used. Schematic shows steps required to read the data into InSituPy, annotate the regions of the individual TMA cores and create an InSituExperiment object using those regions. Subsequently, metadata can be added, HE images can be registered automatically and different preprocessing functions can be applied. After saving the data, it can be also visualized either interactively using the implemented napari viewer or statically using different plotting functions.

Supplementary Tables

Method	Language	Data modalities							Functionalities				
		Multi-sample level	Sample level						Multi-sample level operations and analyses	Automated image registration	Interactive visualization	Spatial trajectory analysis	ROI analysis
		Metadata	Metadata	Images	Transcripts	Single-cell omics data	Spatial Units	Annotations					
SpatialExperiment	R	No	Yes	Yes	Yes	Yes	Yes	Yes	Yes	No	No	No	No
Giotto	R	No	Yes	Yes	Yes	Yes	Yes	Yes	No	No	Yes	No	No
SpatialData	Python	No	Yes	Yes	Yes	Yes	Yes	Yes	No	No	Yes	No	Yes
Squidpy	Python	No	Yes	Yes	No	Yes	Yes	Yes	No	No	Yes	No	No
SPATA2	R	No	Yes	Yes	No	Yes	Yes	Yes	No	No	No	Yes	Yes
VoltRon	R	No	Yes	Yes	Yes	Yes	Yes	Yes	No	Yes	Yes	No	Yes
Voyager	R, Python	No	Yes	Yes	Yes	Yes	Yes	Yes	No	No	No	No	No
ATHENA	Python	Yes	Yes	Yes	No	Yes	Yes	No	No	No	Yes	No	No
MoleculeExperiment	R	No	Yes	Yes	Yes	Yes	No	Yes	Yes	No	No	No	No
InSituPy	Python	Yes	Yes	Yes	Yes	Yes	Yes	Yes	Yes	Yes	Yes	Yes	Yes

Table S1. Comparison of functionalities in different frameworks for spatial omics data.

Modality	Attribute name	Loading function	Data format	
			After loading	On disk
**Single-cell transcriptome**	*.cells.table*	*load_cells()*	AnnData	H5AD
**Cellular segmentation masks**	*.cells.boundaries*		Dask Array	Zarr
**Transcriptomic data of spatial units**	*.units.table*	*load_units()*	AnnData	H5AD
**Geometric shapes of spatial units**	*.units.shapes*		GeoPandas GeoDataFrame	Parquet
**Image data**	*.images*	*load_images()*	Dask Array	Zarr/OME-TIFF
**Transcript locations and identities**	*.transcripts*	*load_transcripts()*	Dask DataFrame	Parquet
**Histological annotations**	*.annotations*	*load_annotations()*	GeoPandas GeoDataFrame	GeoJSON
**Regional annotations**	*.regions*	*load_regions()*	GeoPandas GeoDataFrame	GeoJSON

Table S2. Data formats used for the different data modalities in InSituPy.

Function name	URL
xenium_human_breast_cancer	https://cf.10xgenomics.com/samples/xenium/1.0.1/Xenium_FFPE_Human_Breast_Cancer_Rep1/Xenium_FFPE_Human_Breast_Cancer_Rep1_outs.zip
xenium_human_kidney_nondiseas	https://cf.10xgenomics.com/samples/xenium/1.5.0/Xenium_V1_hKidney_nondiseased_section/Xenium_V1_hKidney_nondiseased_section_outs.zip
xenium_human_pancreatic_cancer	https://cf.10xgenomics.com/samples/xenium/1.6.0/Xenium_V1_hPancreas_Cancer_Add_on_FFPE/Xenium_V1_hPancreas_Cancer_Add_on_FFPE_outs.zip
xenium_human_skin_melanoma	https://cf.10xgenomics.com/samples/xenium/1.7.0/Xeniumranger_V1_hSkin_Melanoma_Add_on_FFPE/Xeniumranger_V1_hSkin_Melanoma_Add_on_FFPE_outs.zip
xenium_human_brain_cancer	https://s3-us-west-2.amazonaws.com/10x.files/samples/xenium/2.0.0/Xenium_V1_Human_Brain_GBM_FFPE/Xenium_V1_Human_Brain_GBM_FFPE_outs.zip
xenium_human_lung_cancer	https://cf.10xgenomics.com/samples/xenium/2.0.0/Xenium_V1_humanLung_Cancer_FFPE/Xenium_V1_humanLung_Cancer_FFPE_outs.zip
xenium_human_lymph_node_5k	https://s3-us-west-2.amazonaws.com/10x.files/samples/xenium/3.0.0/Xenium_Prime_Human_Lymph_Node_Reactive_FFPE/Xenium_Prime_Human_Lymph_Node_Reactive_FFPE
xenium_human_lymph_node	https://cf.10xgenomics.com/samples/xenium/1.5.0/Xenium_V1_hLymphNode_nondiseased_section/Xenium_V1_hLymphNode_nondiseased_section_outs.zip
xenium_test_dataset_v2_mm	https://cf.10xgenomics.com/samples/xenium/2.0.0/Xenium_V1_human_Breast_2fov/Xenium_V1_human_Breast_2fov_outs.zip
xenium_test_dataset_v2_nucex	https://cf.10xgenomics.com/samples/xenium/2.0.0/Xenium_V1_human_Lung_2fov/Xenium_V1_human_Lung_2fov_outs.zip
xenium_test_dataset_v3_mm	https://cf.10xgenomics.com/samples/xenium/3.0.0/Xenium_Prime_MultiCellSeg_Mouse_Ileum_tiny/Xenium_Prime_MultiCellSeg_Mouse_Ileum_tiny_outs.zip
xenium_test_dataset_v3_nucex	https://cf.10xgenomics.com/samples/xenium/3.0.0/Xenium_Prime_Mouse_Ileum_tiny/Xenium_Prime_Mouse_Ileum_tiny_outs.zip
xenium_test_dataset_v4_nucex	https://cf.10xgenomics.com/samples/xenium/4.0.0/Xenium_V1_Human_Ovary_tiny/Xenium_V1_Human_Ovary_tiny_outs.zip
xenium_test_dataset_v4_mm	https://cf.10xgenomics.com/samples/xenium/4.0.0/Xenium_V1_MultiCellSeg_Human_Ovary_tiny/Xenium_V1_MultiCellSeg_Human_Ovary_tiny_outs.zip
xenium_test_dataset_v4_protein	https://cf.10xgenomics.com/samples/xenium/4.0.0/Xenium_V1_Protein_Human_Kidney_tiny/Xenium_V1_Protein_Human_Kidney_tiny_outs.zip
visium_human_breast_cancer	https://cf.10xgenomics.com/samples/spatial-exp/2.0.0/CytAssist_FFPE_Human_Breast_Cancer/CytAssist_FFPE_Human_Breast_Cancer_filtered_feature_bc_matrix.h5

Table S3. Source of example datasets implemented in InSituPy.

dataset name	URL	file name
IPFTMA5	https://www.ncbi.nlm.nih.gov/geo/query/acc.cgi?acc=GSE250346	GSE250346_IPFTMA5.tar.gz
THD0008	https://www.ncbi.nlm.nih.gov/geo/query/acc.cgi?acc=GSM7990532	GSM7990532_output-XETG00048__0003392__THD0008__20230313__191400.tar.gz
VUILD106	https://www.ncbi.nlm.nih.gov/geo/query/acc.cgi?acc=GSM7990548	GSM7990548_output-XETG00048__0003392__VUILD106MA__20230313__191400.tar.g
Janesick_et_al	https://www.10xgenomics.com/products/xenium-in-situ/preview-datase	Xenium Output Bundle
Human_GBM	https://www.10xgenomics.com/datasets/ffpe-human-brain-cancer-data	Xenium Output Bundle (full)

Table S4. Source of example datasets for benchmarking Xenium In Situ readers.

References

Bankhead P, Loughrey MB, Fernández JA *et al.* QuPath: Open source software for digital pathology image analysis. *Sci Rep* 2017;**7**(1):16878. https://doi.org/10.1038/s41598-017-17204-5.

Besson S, Leigh R, Linkert M *et al.* Bringing Open Data to Whole Slide Imaging. *Digit Pathol 15th Eur Congr ECDP 2019 Warwick UK April 10-13 2019 Proc Eur Congr Digit Pathol 15th 2019 Warwick Engl* 2019;**2019**:3–10. https://doi.org/10.1007/978-3-030-23937-4_1.

Gillies S, others. Shapely: manipulation and analysis of geometric objects. toblerity.org, 2007. https://github.com/Toblerity/Shapely.

Janesick A, Shelansky R, Gottscho AD *et al.* High resolution mapping of the tumor microenvironment using integrated single-cell, spatial and in situ analysis. *Nat Commun* 2023;**14**(1):art. 1. https://doi.org/10.1038/s41467-023-43458-x.

Jordahl K. GeoPandas: Python tools for geographic data. *URL Httpsgithub Comgeopandasgeopandas* 2014.

Kuleshov MV, Jones MR, Rouillard AD *et al.* Enrichr: a comprehensive gene set enrichment analysis web server 2016 update. *Nucleic Acids Res* 2016;**44**(Web Server issue):W90–7. https://doi.org/10.1093/nar/gkw377.

Lowe DG. Distinctive Image Features from Scale-Invariant Keypoints. *Int J Comput Vis* 2004;**60**(2):91–110. https://doi.org/10.1023/B:VISI.0000029664.99615.94.

Marconato L, Palla G, Yamauchi KA *et al.* SpatialData: an open and universal data framework for spatial omics. *Nat Methods* 2025;**22**(1):58–62. https://doi.org/10.1038/s41592-024-02212-x.

Miles A, Kirkham J, Durant M *et al. Zarr-Developers/Zarr-Python: V2.4.0*. Zenodo, 11 Jan. 2020. https://doi.org/10.5281/zenodo.3773450.

Muja M, Lowe DG. Fast Approximate Nearest Neighbors with Automatic Algorithm Configuration. *Int Conf Comput Vis Theory Appl* 2009. https://api.semanticscholar.org/CorpusID:7317448.

Otto DJ, Jordan C, Dury B *et al.* Quantifying cell-state densities in single-cell phenotypic landscapes using Mellon. *Nat Methods* 2024;**21**(7):1185–95. https://doi.org/10.1038/s41592-024-02302-w.

Palla G, Spitzer H, Klein M *et al.* Squidpy: a scalable framework for spatial omics analysis. *Nat Methods* 2022;**19**:171–8. https://doi.org/10.1038/s41592-021-01358-2.

Raudvere U, Kolberg L, Kuzmin I *et al.* g:Profiler: a web server for functional enrichment analysis and conversions of gene lists (2019 update). *Nucleic Acids Res* 2019;**47**(W1):W191–8. https://doi.org/10.1093/nar/gkz369.

Rocklin M. Dask: Parallel Computation with Blocked algorithms and Task Scheduling. In: Huff K, Bergstra J (eds), *Proceedings of the 14th Python in Science Conference*. 2015, 130–6.

Sofroniew N, Lambert T, Bokota G *et al. Napari: A Multi-Dimensional Image Viewer for Python*, version v0.5.4. Zenodo, 30 Sept. 2024. https://doi.org/10.5281/zenodo.13863809.

Szklarczyk D, Gable AL, Lyon D *et al.* STRING v11: protein–protein association networks with increased coverage, supporting functional discovery in genome-wide experimental datasets. *Nucleic Acids Res* 2019;**47**(D1):D607–13. https://doi.org/10.1093/nar/gky1131.

Vannan A, Lyu R, Williams AL *et al.* Spatial transcriptomics identifies molecular niche dysregulation associated with distal lung remodeling in pulmonary fibrosis. *Nat Genet* 2025;**57**(3):647–58. https://doi.org/10.1038/s41588-025-02080-x.

Virshup I, Bredikhin D, Heumos L *et al.* The scverse project provides a computational ecosystem for single-cell omics data analysis. *Nat Biotechnol* 10 Apr. 2023:1–3. https://doi.org/10.1038/s41587-023-01733-8.

Virshup I, Rybakov S, Theis FJ *et al.* anndata: Annotated data. *bioRxiv* 19 Dec. 2021:2021.12.16.473007. https://doi.org/10.1101/2021.12.16.473007.

Wolf FA, Angerer P, Theis FJ. SCANPY: Large-scale single-cell gene expression data analysis. *Genome Biol* 2018;**19**(1):15. https://doi.org/10.1186/s13059-017-1382-0.
